# Circle-map profiling of extrachromosomal circular DNA as diagnostic biomarkers for lung cancer

**DOI:** 10.1093/pcmedi/pbae006

**Published:** 2024-03-22

**Authors:** Yongfeng Yang, Tingting Song, Sha Liu, Zhiqiang Liu, Xuehui Wang, Yi Li, Dan Liu

**Affiliations:** Department of Respiratory and Critical Care Medicine, Institute of Respiratory Health, Center of Precision Medicine, West China Hospital, Sichuan University, Chengdu 610041, China; Department of Respiratory and Critical Care Medicine, Institute of Respiratory Health, Center of Precision Medicine, West China Hospital, Sichuan University, Chengdu 610041, China; Department of Respiratory and Critical Care Medicine, Institute of Respiratory Health, Center of Precision Medicine, West China Hospital, Sichuan University, Chengdu 610041, China; Department of Respiratory and Critical Care Medicine, Institute of Respiratory Health, Center of Precision Medicine, West China Hospital, Sichuan University, Chengdu 610041, China; Department of Respiratory and Critical Care Medicine, Institute of Respiratory Health, Center of Precision Medicine, West China Hospital, Sichuan University, Chengdu 610041, China; Department of Respiratory and Critical Care Medicine, Institute of Respiratory Health, Center of Precision Medicine, West China Hospital, Sichuan University, Chengdu 610041, China; Department of Respiratory and Critical Care Medicine, Institute of Respiratory Health, Center of Precision Medicine, West China Hospital, Sichuan University, Chengdu 610041, China

**Dear Editor**:

Lung cancer has the highest incidence rate among malignancies and remains the primary contributor to cancer-related mortality on a global scale [1]. Early detection is the most efficacious approach for enhancing the prognosis and mitigating the mortality of patients with lung cancer [2]. Currently, diagnosis of lung cancer is mainly by different types of imaging including low-dose spiral computed tomography (LDCT) combined with pathological assessment of biopsy, but prevalence of false-positive results and associated expenses may impede its becoming a standard screening technique [3]. Therefore, identifying new reliable biomarkers for early and accurate diagnosis of lung cancer is crucial for identifying effective treatments as therapeutic targets. It is reported that that extrachromosomal circular DNAs (eccDNAs) are abnormally expressed in tumors and could serve as diagnostic markers for cancers [4–7], but it is unclear whether eccDNA in plasma can be used as a potential marker for lung cancer diagnosis. We here identified the expression patterns and clinical implications of plasma eccDNAs of non-small cell lung cancer (NSCLC) patients, revealing the potential of plasma eccDNA as candidate biomarkers for lung cancer diagnosis.

We profiled eccDNA expression in plasma samples of 17 (I:16, II:1) early-stage NSCLC patients, 8 (III:2, IV:6) advanced-stage NSCLC patients and 6 healthy controls via Circle-Map. We also analyzed and compared the characteristic of eccDNAs (the counts, the length and genomic distribution etc.) between healthy controls and NSCLC patients in both early and advanced stages. The differential and consistent eccDNA in patients with early NSCLC was selected as the candidate eccDNA for NSCLC diagnosis. A risk predictive model for lung cancer screening was established through examining consistent eccDNAs in plasma of early lung cancer samples. Receiver operating characteristic (ROC) curve analysis was used to validate the performance of the predictive model.

## Differentially expressed eccDNAs in plasma of healthy controls and lung cancer patients

**Figure 1.**
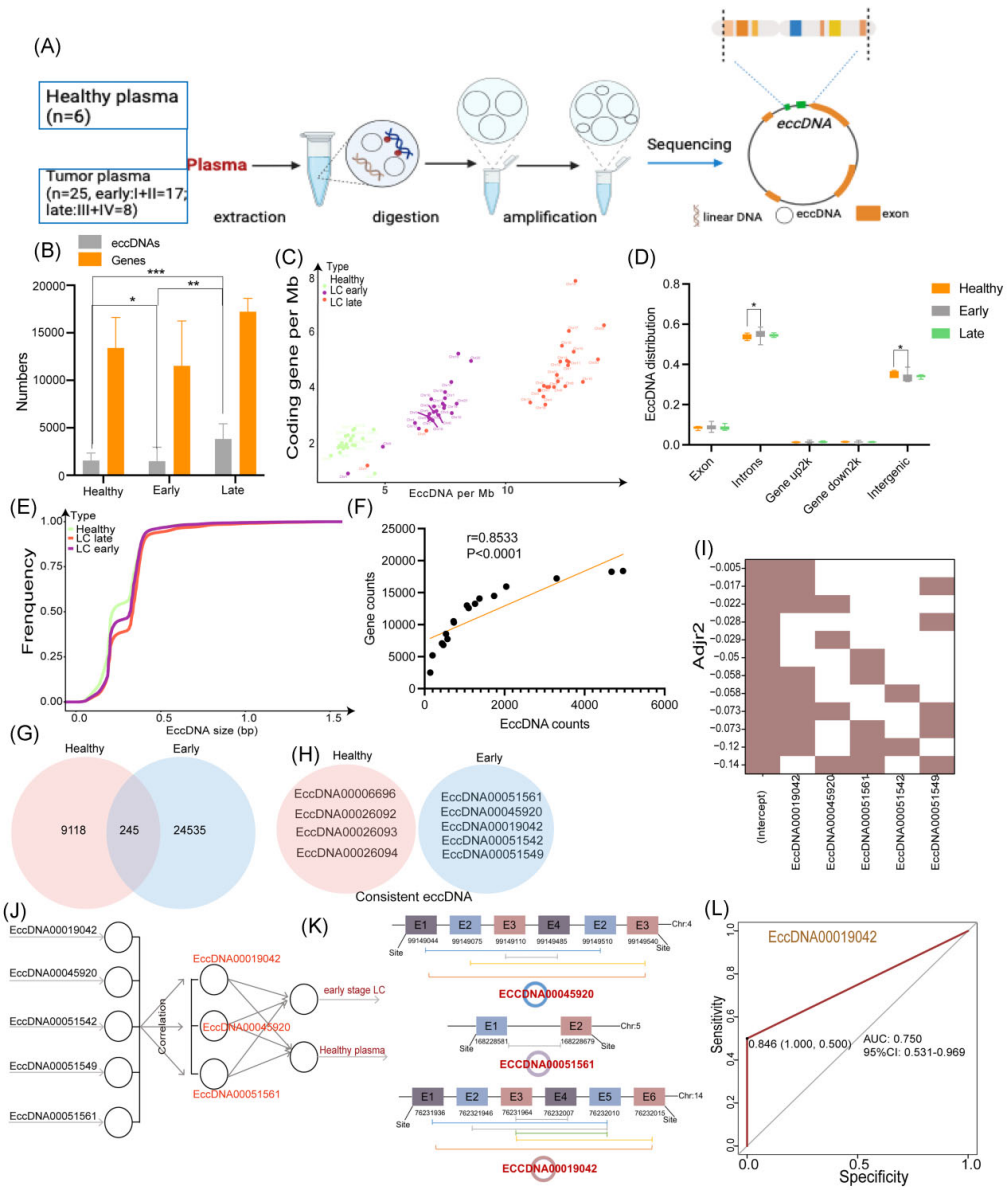
Identifying potential eccDNAs in early NSCLC plasma samples for diagnosis. (A) The workflow of the processes for detecting eccDNAs in plasma. (B) The counts of eccDNAs and eccDNA genes in samples from healthy controls, early stage and late stage lung cancer patients. (C) The ratios of coding genes/Mb and eccDNAs/Mb of chromosomes in samples from healthy controls, early stage and late stage lung cancer patients. (D) Genomic distributions of eccDNAs in samples from healthy controls, early stage and late stage lung cancer patients. (E) Size distribution of eccDNAs in samples from healthy controls, early stage and late stage lung cancer patients. (F) Pearson correlation analysis between the coding gene/Mb and eccDNA/Mb ratios on chromosomes from healthy controls, early stage and late stage lung cancer patients. (G, H) Consistent eccDNAs and consistent eccDNA genes sequences in tissue samples from healthy controls and early stage lung cancer patients. (I) Heatmap showing the distribution of adjusted R2 values in each model, which was calculated by the leaps R package. (J) The selection workflow for important diagnostic eccDNAs to distinguish early NSCLC from healthy plasma samples. (K) The split sites of ECCDNA00045920 and ECCDNA00051561. (L) ROC curves for EccDNA00019042 in the test dataset (ROC analyses of EccDNA00019042 in early NSCLC and normal plasma samples; early AUC = 0.750, 95% CI = 0.531–0.969).

The characteristics of plasma eccDNAs from 6 healthy controls, 17 early stage lung cancer patients (16 of Stage I and 1 of Stage II), and 8 lung cancer patients in advanced stage (2 of Stage III and 6 of Stage IV) were analyzed for diagnosis of lung cancer by the Circle-Seq method [8–10] (Fig. 1A; Tables S1‒S3, see on-line supplementary material). The results showed the tumor plasma sample had more eccDNAs than that from healthy controls (Fig. S1A and B, see on-line supplementary material); in particular, samples from advanced-stage patients had the largest count of eccDNA among the three groups (Fig. 1B). There was no difference in the number of eccDNA-carrying gene fragments. The genomic distribution of the eccDNAs in early lung cancer plasma was similar to that in total plasma samples and was different and comparable among the three groups (Fig. 1C; Fig. S1C, see on-line supplementary material). However, no significant differences existed in the genomic elements of the annotated eccDNAs among the three groups of plasma samples, which were also enriched in introns and intronic regions (Fig. 1D; Fig. S1D, see on-line supplementary material). In terms of the length of plasma eccDNAs, the order was healthy controls < early-stage tumors < advanced-stage tumors (Fig. 1E). There was a positive correlation between the ratio of coding genes/Mb and eccDNA/Mb on all chromosomes (early plasma samples: r = 0.8533, P < 0.001; Fig. 1F; Fig. S1F, see on-line supplementary material).

## Differentially expressed eccDNAs in plasma of healthy controls and patients of early lung cancer

To verify whether the unique eccDNAs in early-stage tumor plasma could be used as early diagnostic biomarkers, we selected consistent eccDNAs and eccDNA-carrying gene fragments. As shown in Fig. 1G and H, five consistent eccDNAs were present only in the early tumor samples (EccDNA00051561, EccDNA00045920, EccDNA00019042, EccDNA00051542, and EccDNA00051549) (Table S4, see on-line supplementary material). Four consistent eccDNAs were present only in the healthy samples (EccDNA00006696, EccDNA00026092, EccDNA00026093 and EccDNA00026094) (Table S5, see on-line supplementary material). Three consistent eccDNA genes were present only in the healthy samples (ENSG00000274167, ENSG00000261978 and ENSG00000167291). Four consistent eccDNA genes were present only in the early plasma samples (ENSG000253978, ENSG000198099, ENSG00089916 and ENSG000145934), and they were significantly different from those in the healthy plasma samples (Table S6, see on-line supplementary material).

## eccDNA as potential biomarkers for NSCLC diagnosis

We used machine learning to further determine the diagnostic potential factors of the above selected eccDNAs. As shown in Fig. 1I, strong correlations existed among EccDNA00019042, EccDNA00045920 and EccDNA00051561. EccDNA00019042 showed the largest adjusted R2 value and thus was selected as an important factor in LASSO regression (Fig. 1J). In addition, EccDNA00019042, EccDNA00045920 and EccDNA00051561 were found in the split site of the chromosome (Fig. 1K). Furthermore, receiver operating characteristic (ROC) curve analysis was performed for the three eccDNAs. The area under the curve (AUC) for discriminating early-stage tumors from normal samples was 0.75 (95% CI: 0.531–0.969) for EccDNA00019042 (Fig. 1L), 0.583 (95% CI: 0.420–0.747) for EccDNA00045920, and 0.583 (95% CI: 0.420–0.747) for EccDNA00051561 (Fig. S1G and H, see on-line supplementary material). Moreover, based on the analysis of eccDNA sequencing results of 3 NSCLC tissues and normal lung tissues in our previous research [10], we found a trend of higher expression of EccDNA00019042 in NSCLC tissues compared with normal lung tissues (Table S7, see on-line supplementary material). These results suggested the high diagnostic potential of EccDNA00019042 for early-stage lung cancer.

In summary, we demonstrated the presence of eccDNAs in plasma and described their characteristics and genomic landscape. Though with distinct expression signatures, eccDNA can be present in lung cancer and healthy human plasma. However, the expression level of eccDNAs showed no significant differences, possibly due to the limited sample size and immature analysis methods. Based on the consistent presence of eccDNAs in early lung cancer plasma samples, we established a model to demonstrate that EccDNA00019042 could serve as a diagnostic biomarker for early-stage lung cancer. However, due to the relatively small clinical sample size, individual variations in eccDNAs among the samples included in this study may have affected the robustness of our findings to a certain extent, emphasizing the need for additional studies with larger sample sizes.

## Supplementary Material

pbae006_Supplemental_File
