# Supplementary material for: Circle-map profiling of extrachromosomal circular DNA as diagnostic biomarkers for lung cancer
Source: Precis Clin Med. 2024 Mar 22;7(1):pbae006. doi: 10.1093/pcmedi/pbae006 (PMC11015151; doi:10.1093/pcmedi/pbae006)
Supplement: pbae006_Supplemental_File [file pbae006_supplemental_file.docx]

**Supplementary materials**

| Characteristics | N(%) |
| --- | --- |
| Total number of patients | 25(100%) |
| Sex |  |
| Female | 12(48%) |
| Male | 13(52%) |
| Age at the diagnosis, years |  |
| Median(Range) | 60.8(43-73) |
| <60 | 9(36%) |
| ≥60 | 16(64%) |
| Smoking |  |
| Current smoker | 8(32%) |
| Former smoker | 1(4%) |
| No | 16(64%) |
| T Stage |  |
| T1 | 20(80%) |
| T2 | 1(4%) |
| T3 | 2(8%) |
| T4 | 2(8%) |
| N Stage |  |
| N0 | 20(80%) |
| N1 | 1(4%) |
| N2 | 3(12%) |
| N3 | 1(4%) |
| Disease Stage |  |
| I (early) | 16(64%) |
| II (early) | 1(4%) |
| III (advanced) | 2(8%) |
| IV (advanced) | 6(24%) |

**Table S1** Patient characteristics.

**Table S2** Detail characteristics of NSCLC patients.

| Sample | Sex | Age | Smoking | Type | stage | Group |
| --- | --- | --- | --- | --- | --- | --- |
| I1 | Male | 60 | No | LUAD | I | early |
| I2 | Female | 64 | No | LUAD | I | early |
| I3 | Female | 54 | No | LUAD | I | early |
| I4 | Male | 65 | No | LUAD | I | early |
| I5 | Male | 69 | No | LUAD | I | early |
| I6 | Female | 55 | No | LUAD | I | early |
| I7 | Male | 61 | Yes | LUAD | I | early |
| I8 | Female | 43 | No | LUAD | I | early |
| I9 | Male | 51 | Yes | LUAD | I | early |
| I10 | Female | 63 | No | LUAD | I | early |
| I11 | Male | 52 | Yes | LUAD | I | early |
| I12 | Female | 62 | No | LUAD | I | early |
| I13 | Female | 59 | No | LUAD | I | early |
| I14 | Female | 61 | No | LUAD | I | early |
| I15 | Female | 72 | No | LUAD | I | early |
| I16 | Male | 54 | Yes | LUAD | I | early |
| II1 | Male | 61 | Yes | LUAD | II | early |
| III1 | Male | 73 | No | LUAD | III | advanced |
| III2 | Male | 59 | Yes | LUAD | III | advanced |
| IV1 | Female | 50 | No | LUAD | IV | advanced |
| IV2 | Male | 65 | Yes | LUAD | IV | advanced |
| IV3 | Male | 64 | Yes | LUAD | IV | advanced |
| IV4 | Female | 65 | No | LUAD | IV | advanced |
| IV5 | Male | 66 | Yes | LUAD | IV | advanced |
| IV6 | Female | 72 | No | LUAD | IV | advanced |

**Table S3** Quality control for sample/Statistics of reads for sample.

| Sample | Raw Data(bp) | BF_Q30(%) | Clean Data(bp) | AF_Q30(%) | eccDNA count |
| --- | --- | --- | --- | --- | --- |
| Healthy1 | 14,244,393,900 | 89.29 | 10,432,064,273 | 93.28 | 536 |
| Healthy2 | 14,244,393,901 | 88.9 | 10,432,064,274 | 93.06 | 1720 |
| Healthy3 | 14,244,393,902 | 87.82 | 10,432,064,275 | 93.26 | 1824 |
| Healthy4 | 14,244,393,903 | 85.96 | 10,432,064,276 | 93.66 | 864 |
| Healthy5 | 14,244,393,904 | 86.88 | 10,432,064,277 | 93.16 | 1665 |
| Healthy6 | 14,244,393,905 | 88.37 | 10,432,064,278 | 93.59 | 2754 |
| I1 | 14,244,393,906 | 89.31 | 10,432,064,279 | 93 | 1101 |
| I2 | 14,244,393,907 | 88.64 | 10,432,064,280 | 93.72 | 4962 |
| I3 | 14,244,393,908 | 89.67 | 10,432,064,281 | 93.7 | 2042 |
| I4 | 14,244,393,909 | 89.80 | 10,432,064,282 | 92.68 | 3300 |
| I5 | 14,244,393,910 | 89.96 | 10,432,064,283 | 93.16 | 725 |
| I6 | 14,244,393,911 | 89.90 | 10,432,064,284 | 93.41 | 1737 |
| I7 | 14,244,393,912 | 86.96 | 10,432,064,285 | 91.82 | 434 |
| I8 | 14,244,393,913 | 89.00 | 10,432,064,286 | 93.55 | 197 |
| I9 | 14,244,393,914 | 90.73 | 10,432,064,287 | 94.14 | 468 |
| I10 | 14,244,393,915 | 88.83 | 10,432,064,288 | 92.31 | 1059 |
| I11 | 14,244,393,916 | 89.86 | 10,432,064,289 | 93.54 | 535 |
| I12 | 14,244,393,917 | 91.42 | 10,432,064,290 | 94.48 | 570 |
| I13 | 14,244,393,918 | 90.08 | 10,432,064,291 | 93.43 | 143 |
| I14 | 14,244,393,919 | 90.19 | 10,432,064,292 | 93.74 | 1262 |
| I15 | 14,244,393,920 | 89.09 | 10,432,064,293 | 93.98 | 732 |
| I16 | 14,244,393,921 | 89.41 | 10,432,064,294 | 93.88 | 1371 |
| II1 | 14,244,393,922 | 88.81 | 10,432,064,295 | 93.53 | 4674 |
| III1 | 14,244,393,923 | 87.09 | 10,432,064,296 | 93.48 | 3946 |
| III2 | 14,244,393,924 | 87.73 | 10,432,064,297 | 93.21 | 4036 |
| IV1 | 14,244,393,925 | 91.76 | 10,432,064,298 | 93.82 | 3460 |
| IV2 | 14,244,393,926 | 88.48 | 10,432,064,299 | 93.28 | 5422 |
| IV3 | 14,244,393,927 | 88.51 | 10,432,064,300 | 93.19 | 1908 |
| IV4 | 14,244,393,928 | 88.82 | 10,432,064,301 | 93.17 | 1457 |
| IV5 | 14,244,393,929 | 88.91 | 10,432,064,302 | 93.77 | 4055 |
| IV6 | 14,244,393,930 | 86.89 | 10,432,064,303 | 93.14 | 6275 |

| EccDNA | Chromosome | Start | End | Sample | Group | Annotation | Symbol |
| --- | --- | --- | --- | --- | --- | --- | --- |
| EccDNA00051561 | 5 | 168228581 | 168228679 | I3,I5,II1 | early NSCLC | ENSG00000253978 | - |
| EccDNA00045920 | 4 | 99149044 | 99149540 | I5,I2,II1 | early NSCLC | ENSG00000198099 | ADH4 |
| EccDNA00019042 | 14 | 76231936 | 76232015 | I5,II1,I2,NT2,I6,I3 | early NSCLC | ENSG00000089916 | GPATCH2L |
| EccDNA00051542,EccDNA00051549,EccDNA00051561 | 5 | 167813281 | 167813468 | II1,I3,I5 | early NSCLC | ENSG00000145934 | TENM2 |

**Table S4** Consistent eccDNAs in early NSCLC.

| EccDNA | Chromosome | Start | End | Sample | Group | Annotation | Symbol |
| --- | --- | --- | --- | --- | --- | --- | --- |
| EccDNA00006696 | 10 | 38485565 | 42301884 | Healthy6,Healthy3,Healthy2,Healthy5 | Healthy | ENSG00000274167 | - |
| EccDNA00026092,EccDNA00026093,EccDNA00026094 | 17 | 80021762 | 80027504 | Healthy3,Healthy5,Healthy6,Healthy2 | Healthy | ENSG00000261978 | - |
| EccDNA00026092,EccDNA00026093,EccDNA00026094 | 17 | 80021762 | 80027504 | Healthy3,Healthy5,Healthy6,Healthy2 | Healthy | ENSG00000167291 | TBC1D16 |

**Table S5** Consistent eccDNAs in healthy group.

**Table S6** Higher expression of consistent eccDNAs in early NSCLC plasma than in healthy controls.

| EccDNA | Chromosome | Start | End | Healthy mean | early NSCLC mean | log2 (fc) | P Value | Annotation | Symbol |  | Advanced NSCLC mean |
| --- | --- | --- | --- | --- | --- | --- | --- | --- | --- | --- | --- |
| EccDNA00019042 | 14 | 76231936 | 76232015 | 0.001 | 93.07838118 | 16.5061585 | 0.436392121 | ENSG00000089916 | GPATCH2L | ↑ | 0.001 |
| EccDNA00045920 | 4 | 99149044 | 99149540 | 0.001 | 245.7795343 | 17.90700526 | 0.598655063 | ENSG00000246090,ENSG00000198099 | -,ADH4 | ↑ | 0.001 |
| EccDNA00051542 | 5 | 167601753 | 167602094 | 0.001 | 0.001 | 0 | 1 | ENSG00000145934 | TENM2 | ↑ | 0.001 |
| EccDNA00051549 | 5 | 167813281 | 167813468 | 0.001 | 0.001 | 0 | 1 | ENSG00000145934 | TENM2 | ↑ | 0.001 |
| EccDNA00051561 | 5 | 168228581 | 168228679 | 0.001 | 84.7258722 | 16.37051496 | 0.658238873 | ENSG00000253978,ENSG00000145934 | -,TENM2 | ↑ | 0.001 |

**Table S7** Higher expression of consistent eccDNAs in early NSCLC tissues than in healthy controls.

| EccDNA | Chromosome | Start | End | NT mean | LT mean | log2 (fc) | P Value | Annotation | Symbol |  |
| --- | --- | --- | --- | --- | --- | --- | --- | --- | --- | --- |
| EccDNA00001299 | 14 | 76231964 | 76232010 | 0.001 | 395.7 | 18.59 | 0.3739 | ENSG00000089916 | GPATCH2L | ↑ |


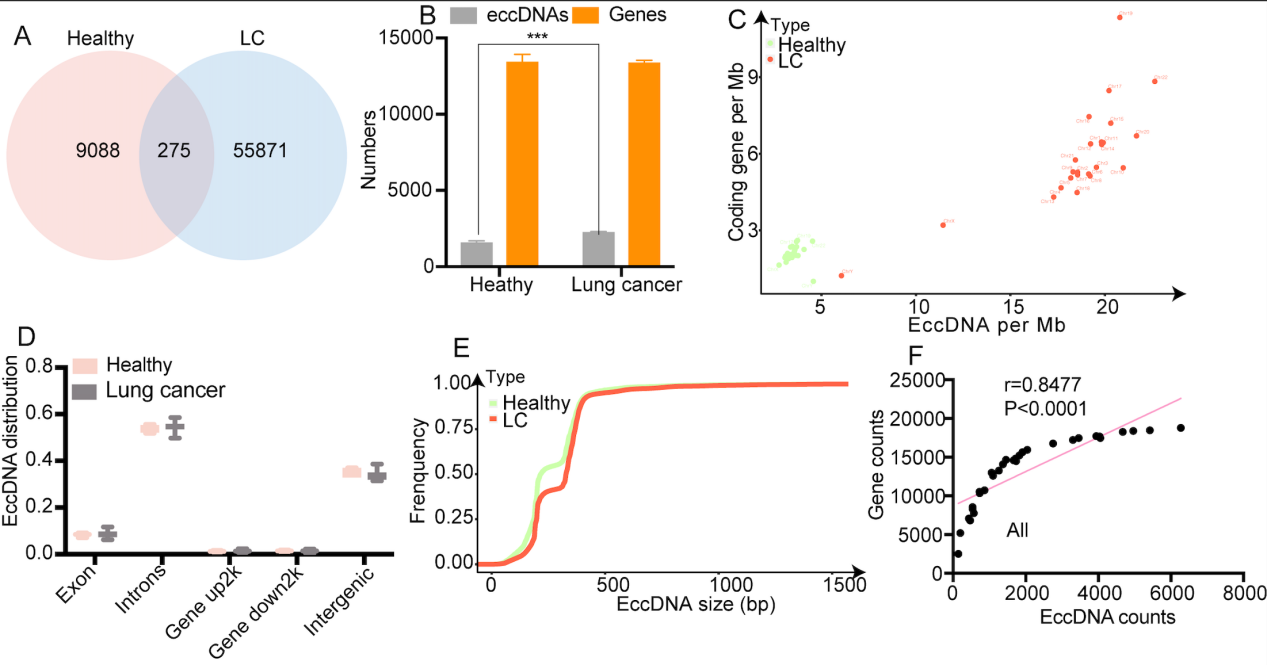


**Figure S1.** Identifying eccDNAs in NSCLC plasma samples. (A, B) The counts of eccDNAs and eccDNA genes in two groups. (C) The ratios of coding genes/Mb and eccDNAs/Mb of chromosomes in all groups. (D) Genomic distributions of eccDNAs in two group samples. (E) Size distribution of eccDNAs in two groups. (F) Pearson correlation analysis between the coding gene/Mb and eccDNA/Mb ratios on any of the chromosomes in two groups.

**Materials and methods**

**Clinical sample collection**

This study was approved by the Ethics Committee of West China Hospital of Sichuan University, and written informed consent was obtained from the patients. Samples were collected following the relevant ethical regulations for human participants. Patient admission criteria were used to identify patients with LUAD at different stages based on pathological staining results. Preparation of plasma from blood by low-speed centrifugation was performed as previously described. Early-stage NSCLC patient plasma (17, I:16, II:1), advanced-stage NSCLC plasma (8, III:2, IV:6) and 6 healthy plasma samples (n=6) were collected from West China Hospital to conduct circle-seq. The detailed clinical characteristics of the patients enrolled in this study are summarized in Table 1.

**EccDNA enrichment and eccDNA sequencing**

High-throughput eccDNA sequencing and subsequent bioinformatics analyses were performed by Genedenovo Biotech, Inc. (Guangzhou, China). eccDNA enrichment, purification and sequencing were performed as described in a previous study (PMID:29540679; PMID:37125628). Specifically, tissue cells were suspended in L1 solution (Plasmid Mini AX; A&A Biotechnology, Pomorskie Gdynia, Poland) supplemented with proteinase K (Thermo Fisher, MA, USA) before being incubated overnight at 50 °C with agitation. After lysis, the samples were treated with an alkaline solution, followed by the precipitation of proteins and separation of chromosomal DNA from circular DNA through an ion exchange membrane column (Plasmid Mini AX; A&A Biotechnology). The remaining linear DNA was removed by exonuclease (Plasmid-Safe ATP-dependent DNase, Epicenter, Wisconsin, USA) at 37 °C in a heating block, and the enzyme reaction was carried out continuously for 1 week, with the application of additional ATP and DNase every 24 hours (30 units per day) according to the manufacturer’s protocol (Plasmid-Safe ATP-dependent DNase, Epicenter). eccDNA-enriched samples were used as templates for phi29 polymerase amplification reactions (REPLI-g Midi Kit), in which eccDNA was amplified at 30 °C for 2 days (46–48 hours). Phi29-amplified DNA was sheared by sonication (Diagenode Bioruptor, NJ, Bioruptor), and the fragmented DNA was subjected to library preparation with the NEBNext® Ultra II DNA Library Prep Kit for Illumina (New England Biolabs). Sequencing was carried out on an Illumina NovaSeq 6000 platform in 150 bp paired-end mode according to the manufacturer’s instructions (PMID:30423086; PMID:19451168; PMID:20110278).
